# Application of High-Frequency Conductivity Map Using MRI to Evaluate It in the Brain of Alzheimer's Disease Patients

**DOI:** 10.3389/fneur.2022.872878

**Published:** 2022-05-16

**Authors:** Soonchan Park, Sue Min Jung, Mun Bae Lee, Hak Young Rhee, Chang-Woo Ryu, Ah Rang Cho, Oh In Kwon, Geon-Ho Jahng

**Affiliations:** ^1^Department of Radiology, College of Medicine, Kyung Hee University Hospital at Gangdong, Kyung Hee University, Seoul, South Korea; ^2^Department of Biomedical Engineering, Undergraduate School, College of Electronics and Information, Kyung Hee University, Yongin-si, South Korea; ^3^Department of Mathematics, College of Basic Science, Konkuk University, Seoul, South Korea; ^4^Department of Neurology, College of Medicine, Kyung Hee University Hospital at Gangdong, Kyung Hee University, Seoul, South Korea; ^5^Department of Psychiatry, College of Medicine, Kyung Hee University Hospital at Gangdong, Kyung Hee University, Seoul, South Korea

**Keywords:** Alzheimer's disease, gray matter volume, MRI, high-frequency conductivity, brain

## Abstract

**Background:**

The previous studies reported increased concentrations of metallic ions, imbalanced Na+ and K+ ions, and the increased mobility of protons by microstructural disruptions in Alzheimer's disease (AD).

**Purpose:**

(1) to apply a high-frequency conductivity (HFC) mapping technique using a clinical 3T MRI system, (2) compare HFC values in the brains of participants with AD, amnestic mild cognitive impairment (MCI), and cognitively normal (CN) elderly people, (3) evaluate the relationship between HFC values and cognitive decline, and (4) explore usefulness of HFC values as an imaging biomarker to evaluate the differentiation of AD from CN.

**Materials and Methods:**

This prospective study included 74 participants (23 AD patients, 27 amnestic MCI patients, and 24 CN elderly people) to explore the clinical application of HFC mapping in the brain from March 2019 to August 2021. We performed statistical analyses to compare HFC maps between the three participant groups, evaluate the association of HFC maps with Mini-Mental State Examination (MMSE) scores, and to evaluate the differentiation between the participant groups for HFC values for some brain areas.

**Results:**

We obtained a good HFC map non-invasively. The HFC value was higher in the AD group than in the CN and MCI groups. MMSE scores were negatively associated with HFC values. Age was positively associated with HFC values. The HFC value in the insula has a high area under the receiver operating characteristic (ROC) curve (AUC) value to differentiate AD patients from the CN participants (Sensitivity [*SE*] = 82, Specificity [*SP*] =97, *AUC* = 0.902, *p* < 0.0001), better than gray matter volume (GMV) in hippocampus (*SE* = 79, SP = 83, *AUC* = 0.880, *p* < 0.0001). The classification for differentiating AD from CN was highest by adding the hippocampal GMV to the insular HFC value (*SE* = 87, *SP* = 87, *AUC* = 0.928, *p* < 0.0001).

**Conclusion:**

High-frequency conductivity values were significantly increased in the AD group compared to the CN group and increased with age and disease severity. HFC values of the insula along with the GMV of the hippocampus can be used as an imaging biomarker to improve the differentiation of AD from CN.

## Summary statement

High-frequency conductivity (HFC) values in the brain are significantly increased in Alzheimer's disease (AD) patients compared to cognitively normal (CN) elderly people, are negatively associated with Mini-Mental State Examination (MMSE) scores, and therefore, can be used as an imaging biomarker to improve the differentiation of AD from CN.

## Key results

1. We obtained a good high-frequency conductivity (HFC) map non-invasively.
2. The HFC values in the brain were higher in Alzheimer's disease (AD) than those with cognitively normal (CN) elderly people and patients with amnestic mild cognitive impairment (MCI).
3. The HFC values negatively associated with Mini-Mental State Examination (MMSE) scores.
4. The addition of insular HFC values to hippocampal gray matter volume (GMV) measurements improved the classification for differentiating AD from CN

## Introduction

Alzheimer's disease (AD) is the most common neurodegenerative disease and is characterized by the progressive loss of memory and other cognitive functions. The neuropathology of AD is generally characterized by the presence of two abnormal proteins in the aggregated state in the brain, intracellular neurofibrillary tangles, and extracellular amyloid-beta (Aβ) plaques. Mild cognitive impairment (MCI) is a high-risk condition for the development of clinically probable AD or other neurological conditions (1). In particular, the amnestic subtype of MCI, which manifests as memory complaints, is often caused by degenerative etiologies and is generally regarded as a precursor of AD (2). A previous study revealed that 16% of amnestic MCI (aMCI) patients progress to dementia each year, 99% of whom receive an AD diagnosis (3). Therefore, the assessment of MCI is beneficial in terms of early AD intervention and perhaps of AD prevention (4). The previous studies showed increased metallic ions (5, 6) and imbalanced Na+ and K+ ions in AD (7, 8). Changes in the ion concentrations can be caused by homeostasis alterations in AD patients. Furthermore, several previous studies showed increased diffusivity caused by demyelination and neurodegeneration in AD patients (9–11), indicating the increased mobility of protons by microstructural disruptions in AD compared to cognitively normal (CN) elderly controls.

Conductivity values vary according to water content, cell shape and size, the mobility of ions and cell membranes, and pathological conditions (12, 13). The conductivity (σ) mapping of human brain tissue could be used as an imaging biomarker for diagnosing diseases and predicting treatment effects. Magnetic resonance electrical property tomography (MREPT) is a technique to derive *in vivo* internal electrical conductivity using a standard MRI system without applying externally mounted electrodes or currents (14, 15). In MREPT, the radiofrequency (RF) magnetic field (B1) of the MRI scanner is used to apply a sinusoidal current to a human brain at the Larmor frequency, which is ~128 MHz for a 3 Tesla clinical MRI system. The current applied by RF pulse induces a time-harmonic magnetic field that is influenced by the brain's admittivity distribution *via* Maxwell's equations. Using the positively rotating magnetic field measured by mapping the B1 field, the electrical conductivity can be extracted (16). Therefore, this conductivity is called Larmor frequency conductivity or high-frequency conductivity (HFC), which is frequency-dependent tissue bulk conductivity. High-frequency current tends to pass through the cell membranes, and low-frequency current is blocked by the cell membrane. However, the conductivity at lower frequency (<1 KHz) cannot be obtained with a clinical MRI scanner without injecting an external current. As HFC imaging is a non-invasive method, various clinical studies have been conducted (17–19).

Conductivity values may provide clinically useful information for neuronal degeneration. In this study, we tried to optimize the MREPT processing step to map HFC and apply it to patients with dementia to evaluate the potential diagnostic value for AD. The previous studies reported statistically significant increases of Na+ in frontal (25%) and parietal cortex (20%), and K+ in cerebellum (15%) and increased concentrations of metallic ions (7, 8). Furthermore, it was reported that diffusivity of water by microstructural disruptions in AD patients is increased compared to CN elderly controls (9–11). The apparent total ion concentration distribution is a component of the conductivity. Hence, we can naturally hypothesize that HFC could be increased in the brain of patients with AD. Furthermore, increasing Aβ and tau proteins in the AD brain can modify neuronal cells, altering the conductivity values in the voxel. Therefore, the purposes of this study were to compare the HFC values in the brain of participants with AD, amnestic MCI, and CN elderly people and evaluate the relationship between HFC values and cognitive decline.

## Materials and Methods

### Participants

Our Institutional Review Board (IRB) approved this cross-sectional prospective study and informed consent was obtained from the participants who were recruited in the neurological center of our institution. Participants provided a detailed medical history and underwent a neurologic examination, standard neuropsychological testing, and MRI scan. The brain MR images of each participant were evaluated by neuroradiologists with more than 15 years of imaging experience to determine any evidence of prior cortical infarctions or other space-occupying lesions.

Cognitive function was assessed using Mini-Mental State Examination (MMSE), and based on the results of the neuropsychological examination and MRI findings, the patients with mild and probable AD were included in this study, defined as those having clinical dementia rating (CDR) scores of 0.5, 1, or 2 according to the criteria of the National Institute of Neurological and Communicative Disorders and the Stroke-Alzheimer's Disease and Related Disorders Association (20, 21). In addition, participants with amnestic MCI, either single-domain or multiple-domain, were also included according to the Petersen criteria (1, 22). Finally, elderly CN participants were selected from healthy volunteers who did not have a medical history of neurological disease, who showed normal results on detailed examination according to the Korean normative database, and who also had a normal brain MRI.

A total of 85 participants were enrolled in this study. Eleven participants were excluded from the subsequent analysis due to the following reasons: two participants had multiple infarctions, one participant had a brain tumor, one participant had a small traumatic subarachnoid hemorrhage lesion, two participants had severe hydrocephalus, one participant had a venous anomaly lesion, two participants had non-amnestic MCI, and two participants had metallic artifacts in the images. Therefore, the 74 remaining participants were grouped as 24 CN, 27 MCI, and 23 AD. Table 1 summarizes the demographic characteristics and the neuropsychological tests of the participant groups. There were several comorbid conditions of the participants in this study. A total of 33 subjects (44.6%) had one or more underlying health conditions and the most common comorbidity was hypertension (29.7%) in all groups. In the AD group with 23 participants, 7 subjects (30.4%) had hypertension and other comorbidities included in diabetes mellitus (2), unruptured intracranial cerebral aneurysm (1), chronic kidney disease (1), myelodysplastic syndrome (1), and transplanted liver (1). In the MCI group with 27 participants, 10 subjects (37%) had hypertension that is also the most common comorbid condition and other comorbidities were diabetes mellitus (5), unruptured aneurysm of the internal carotid artery (1), and ankylosing spondylitis (1). In the normal control group with 24 participants, 5 subjects (20.8%) had hypertension and 5 subjects had diabetes mellitus that was followed by chronic kidney disease (1), nephrotic syndrome (1), atrial fibrillation (1), dilated cardiomyopathy (1), and unruptured aneurysm of the internal carotid artery (1) as underlying conditions.

**Table 1:** Comparisons of the demographic data and result of the neuropsychological tests among the three participant groups.

**Group**	**CN (1)**	**MCI (2)**	**AD (3)**	***p*** **-value (*Post-hoc*)**
No. of participants	24	27	23	Total 74
**Demographic data**				
*Age (year)	73.0 ± 4.7	74.2 ± 4.4	76.1 ± 7.6	*F* = 1.72, *P* = 0.19
#Sex (Male%/Female%)	12/12 (50/50)	19/18 (33.3/66.7)	6/17 (26.1/73.9)	χ^2^ <1.404 *P* > 0.236
*MMSE	27.8 ± 2.3	26.8 ± 1.2	19.6 ± 4.2	*F* = 61.6, *P* <0.001 (1, 3), (2, 3)
CDR (range)	0 (0–0.5)	0.5 (0.5)	1 (0.5–2)	N/A

* *P-value by ANOVA with Scheffé test for the post-hoc test*.

# *P-value by the chi-squared test*.

*Age and Mini-Mental State Examination (MMSE) scores are presented as the mean ± standard deviation. Clinical Dementia Rating (CDR) scores are presented as the median (range) value. Results of the post-hoc test in the parentheses are explained as the followings: significant difference among the three subject groups as (1, 2, 3), between CN and AD as (1, 3), between CN and MCI as (1, 2), and between MCI and AD (2, 3)*.

### MRI Acquisition

MRI was performed using a 3.0 Tesla MRI system equipped with a 32-channel sensitivity encoding head coil (Ingenia, Philips Medical System, Best, The Netherlands). For the brain MREPT images, a multi-echo turbo spin-echo pulse sequence was used (23). Before the data acquisition, we applied a volume shimming method with the volume defined to cover the brain region. The constant level appearance technique was used to achieve homogeneity correction by using coil sensitivity maps acquired in a reference scan. The imaging parameters were as follows: repetition time (*TR)* = 3,200 ms, first echo time (*TE)* = 12 ms with 12 ms intervals, flip angle (*FA*) = 90°, number of echoes (*NE*) = 6, number of average (NSA) = 1, slice thickness = 5 mm, number of slices = 20 without a gap between the slices, slice orientation = transverse, fold-over direction = anterior-posterior (AP), fat shift direction = left, acquired voxel size = 2 mm^3^ × 2 mm^3^ × 5 mm^3^, reconstructed voxel size = 1 mm^3^ × 1 mm^3^ × 5 mm^3^, acquisition matrix = 112 × 112, reconstruction matrix = 224 × 224, field-of-view (FOV) = 224 mm^2^ × 224 mm^2^, SENSE factor = 0, TSE factor = 6, RF shim = “adaptive,” B0 shim = “PB-volume,” slice scan order = interleaved, and regional saturation slab = 45 mm at the feet direction. The scan time of the MREPT sequence was 6 min and 5 s. Real and imaginary images were saved for reconstructing the conductivity map. For image registration and brain tissue segmentation, a sagittal structural three-dimensional (3D) T1-weighted (T1W) image was acquired with the fast field-echo (FFE) sequence, which is similar to the magnetization-prepared rapid acquisition of the gradient echo (MPRAGE) sequence. The imaging parameters were as follows: *TR* = 8.1 ms, *TE* = 3.7 ms, *FA* = 8°, *FOV* = 236 mm^2^ × 236 mm^2^, and voxel size = 1 mm^3^ × 1 mm^3^ × 1 mm^3^. T2-weighted turbo-spin-echo, fluid-attenuated inversion recovery (FLAIR), and gradient-echo images were also acquired to evaluate any brain abnormalities.

### Reconstruction of the HFC Map in the Brain

A home-made software was used to map the HFC at the Larmor frequency of 128 MHz at 3T (14). The MREPT formula based on a convection reaction equation was derived by adding the regularization coefficient (24). To solve the convection reaction partial differential equation, we used the 2-dimensional finite-difference method. Detailed information for reconstructing the HFC map was described in the reconstruction part in the Supplementary Material.

### Post-processing of MR Images

To evaluate HFC maps among the three participant groups, the post-processing was performed using the Statistical Parametric Mapping version 12 (SPM12) software (http://www.fil.ion.ucl.ac.uk/spm/software/spm12/). First, the 3D T1W image and the real MREPT image for each participant were co-registered. Second, the 3D T1W image was spatially normalized into our AD-specific brain template and segmented into gray matter and white matter using the computational anatomy toolbox (CAT12) tool (http://www.neuro.uni-jena.de/cat/). Third, the HFC map was then spatially normalized into the brain template using the deformation field information of the 3D T1W image. Finally, the Gaussian smoothing using the full-width at half maximum (FWHM) of 10 mm^3^ × 10 mm^3^ × 10 mm^3^ was applied to the HFC map as well as brain tissue volumes for voxel-based statistical analyses.

### Statistical Analyses

#### Demographic Data and Neuropsychological Test

Demographic data and other parameters were compared between the three participant groups. Age and MMSE scores were compared between the participant groups using the one-way analysis of variance (ANOVA) test and the Scheffé *post-hoc* test. The values of the neuropsychological test were compared using the Kruskal–Wallis test and the Conover *post-hoc* test because those values were not normally distributed according to the Kolmogorov–Smirnov test (*p* < 0.05). Sex was analyzed using the chi-squared test.

#### Voxel-Based Analyses of HFC and Brain Tissue Volume Maps

Statistical analyses of the HFC and brain tissue volume maps were performed using both voxel-based and region-of-interest (ROI)-based methods. For the voxel-based analysis, first, the HFC maps were compared between the three participant groups using the voxel-wised full factorial design of one-way analysis of covariance (ANCOVA) with age as a covariate. The gray matter volume (GMV) and white matter volume (WMV) maps were also analyzed using the voxel-based ANCOVA with age and total intracranial volume (TIV) as covariates. Second, the HFC maps from all participants were evaluated to identify the association of ages or MMSE scores with age as a covariate using voxel-based multiple regression analysis. The GMV and WMV maps were also analyzed using the voxel-based multiple regression to evaluate the association of ages with TIV as covariate and the association of MMSE scores with age and TIV as covariates. For the voxel-based analyses, a significance level of α = 0.05 was applied with correction for multiple comparisons using the false-discovery rate (FDR) method and clusters with at least 100 contiguous voxels. These voxel-based analyses were performed to define some brain areas as ROIs.

#### Region-of-Interest-Based Analyses of HFC and Brain Tissue Volume Values

For the ROI-based analysis, atlas-based ROIs were defined at the bilateral hippocampus, insula, precuneus, and middle temporal gyrus (MTG) using wfu_pickAtlas software (https://www.nitrc.org/projects/wfu_pickatlas/). Those areas were AD signature regions, significantly different regions of HFC and/or gray matter volume among the three participant groups, and/or significant association regions between MRI measures and ages and/or MMSE scores. ROIs were listed in Supplementary Tables. The HFC, GMV, and WMV values were extracted from the selected ROIs using the Marsbar tool (http://marsbar.sourceforge.net) for the following statistical analyses. First, the Kruskal–Wallis test was used to evaluate the differences in the values between the three participant groups. Second, rank correlation analysis was performed to evaluate the degree of the relationship between the ROI values and age for each ROI. Furthermore, partial correlation analysis was performed to evaluate the degree of the relationship between the ROI values and MMSE scores adjusting for age because the ROI values were associated with age. Finally, a ROC curve analysis was used to evaluate the differentiation between the two participant groups for each MRI measure for each ROI. An additional ROC curve analysis was performed to evaluate the improvement of group classifications by adding brain tissue volume to the HFC values. To do this, the logistic regression analysis was performed to calculate the predicted probabilities using the dependent variable of the participant group and the independent variables of both GMV and/or WMV values of the hippocampus and HFC values of the insula. ROC curve analysis was performed again using the predicted probabilities to evaluate adding the HFC value to the GMV and/or WMV value. In the ROI analysis, α < 0.05 was used to determine the significance level. The statistical analyses were performed using the MedCalc version 19.4 (MedCalc Software Ltd, Ostend, Belgium) statistical program.

## Results

### Participant Characteristics

The ANOVA test showed that the age was not significantly different between the participant groups (*F* = 1.72, *p* = 0.19). The Kruskal–Wallis test showed that the MMSE scores (*F* = 61.6, *p* < 0.001) were significantly different between AD and other groups, as expected. The sex was not significantly different between the participant groups (χ^2^ <1.404, *p* > 0.236). The results of the statistical analysis of the participants' demographic data and the neuropsychological test are summarized in Table 1.

**Figure 1:**
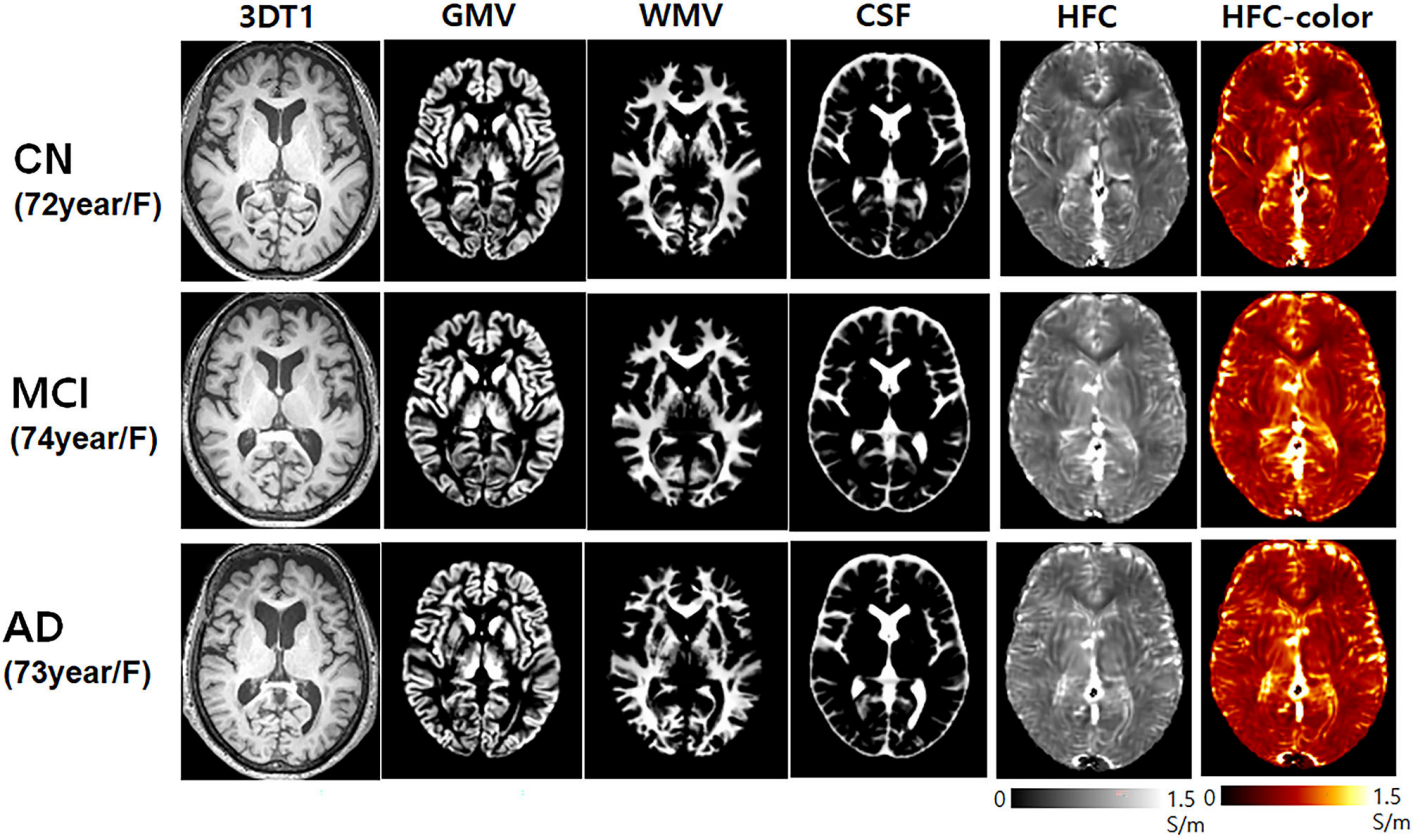
Representative high-frequency conductivity (HFC) maps and the 3D T1-weighted image with the corresponding segmented brain tissue volumes of gray matter and white matter obtained from one cognitively normal (CN) elderly (72-year-old female), one mild cognitive impairment (MCI) (74-year-old female), and one Alzheimer's disease (AD) (73-year-old female) participants.

Figure 1 shows the representative HFC maps with segmented brain tissues obtained from one CN participant (72-year-old female), one mild cognitive impairment (MCI) patient (74-year-old female), and one AD patient (73-year-old female). The HFC signal exhibits increased HFC in the AD patient compared with the CN participant and the MCI patient at the bilateral peri-insular temporal lobe and white matter near the lateral ventricle in the temporal lobe. In general, the HFC signal was high around the parietal lobe, insular, and cerebrospinal fluid (CSF) areas.

### Voxel-Based Analysis of HFC and Brain Tissue Volume Maps

#### Group Comparison

**Figure 2:**
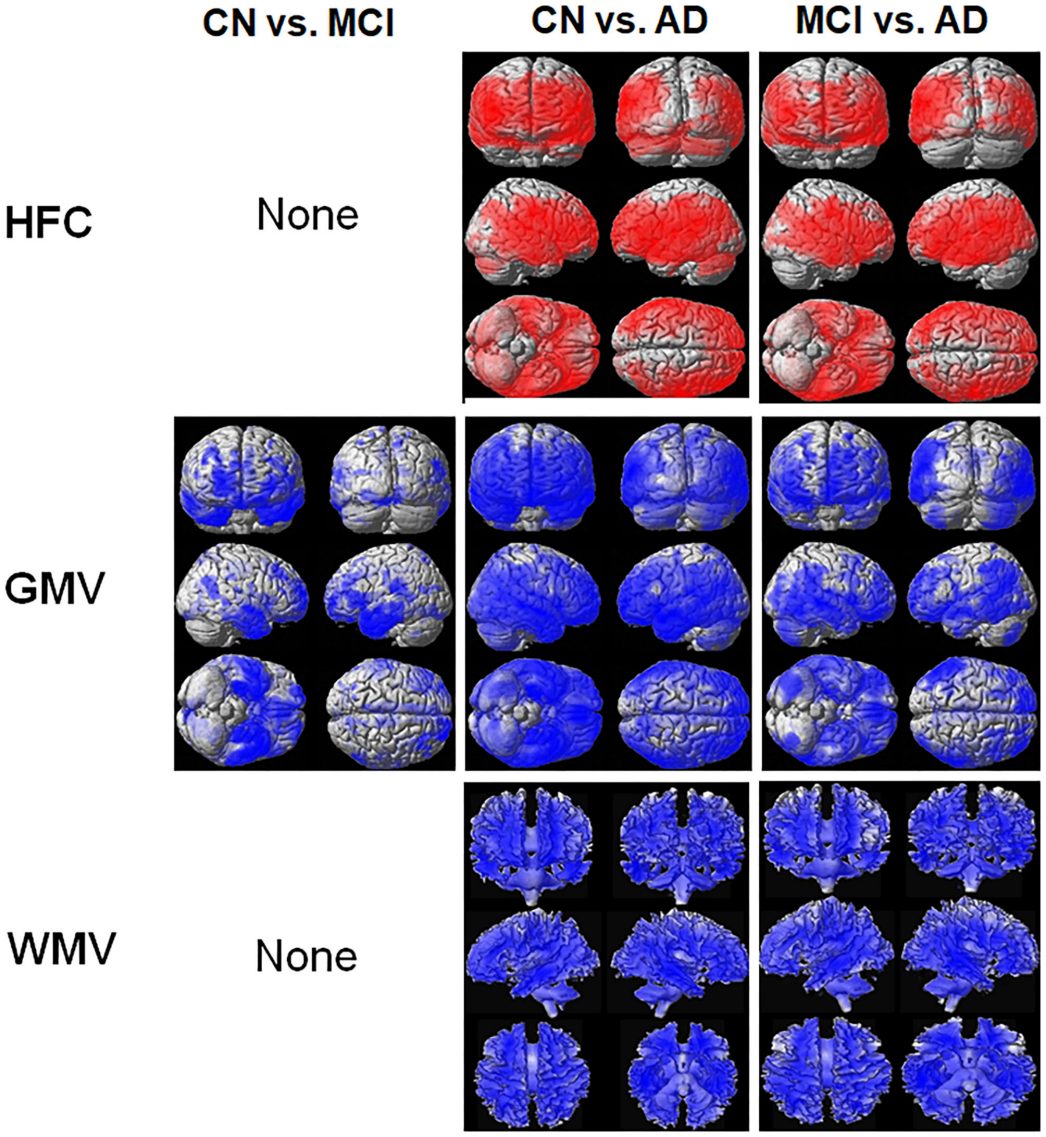
Result of the voxel-based analysis of covariance (ANCOVA) analysis of the HFC and brain tissue maps between the three participant groups. The red color indicates the results of CN < MCI, CN < AD, and MCI < AD and the blue color indicates the results of CN > MCI, CN > AD, and MCI > AD. Details of the significantly different locations in the brain are listed in Supplementary Table 1 for HFC, Supplementary Table 2 for gray matter volume (GMV), and Supplementary Table 3 for white matter volume (WMV).

Figure 2 shows the results of the voxel-based ANCOVA analysis of the HFC and brain tissue maps among the three participant groups. The HFC values were also lower in the CN and MCI groups compared to the AD group at most of brain areas such as in the frontal lobe, the parietal lobe, the temporal lobe, the insula, and precuneus areas (Supplementary Table 1). The brain tissue volume of the GMV (Supplementary Table 2) and WMV (Supplementary Table 3) was higher in the CN and MCI participants than in the AD patients.

#### Association With Age or MMSE Scores

**Figure 3:**
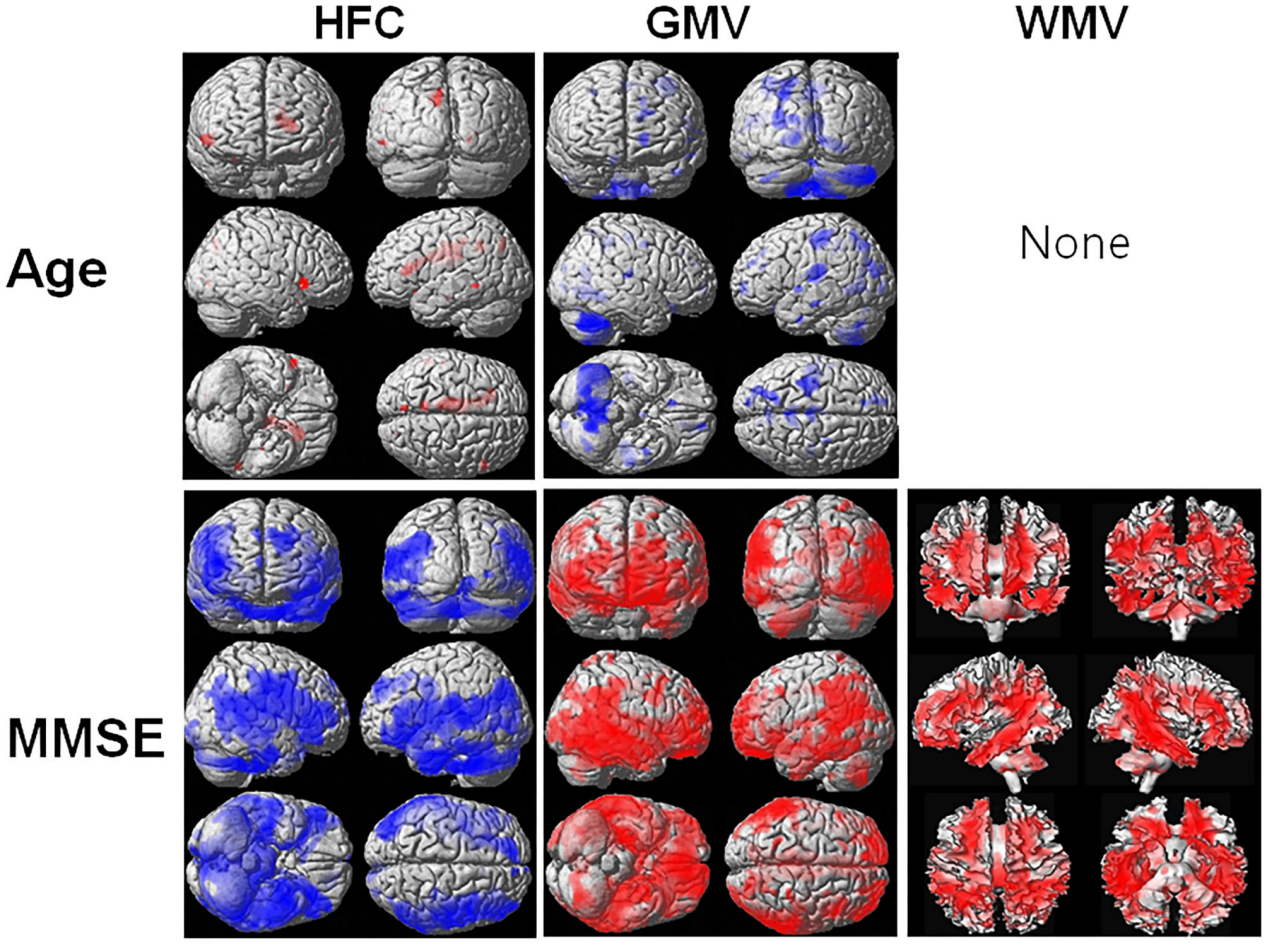
Result of the voxel-based multiple regression analysis of the HFC or brain tissue maps with age or Mini-Mental State Examination (MMSE) scores using all participant data. The blue color indicates a negative association and the red color indicates a positive association. Details of the significantly associated areas in the brain are listed in Supplementary Table 4 with ages and Supplementary Table 5 with MMSE scores.

Figure 3 shows the results of the voxel-based multiple regression analysis of the HFC or brain tissue maps with age or MMSE scores using all participant data. Age was positively associated with HFC values, but negatively associated with GMV (Supplementary Table 4). We did not find any association between age and WMV. The MMSE scores were negatively associated with HFC, but positively associated with GMV and WMV (Supplementary Table 5).

### ROI-Based Analysis of HFC and Brain Tissue Volume Values

#### Group Comparison

Table 2 lists the results of the group comparison for each value and for each ROI. The HFC value was significantly increased in the AD group compared to the CN group in most ROIs. GMV and WMV were significantly decreased in the AD group, as expected.

**Table 2:** Results of comparisons of MRI measures among the three participants groups in the specific brain areas.

**ROI**	**MRI measures**	**CN (1)**	**MCI (2)**	**AD (3)**	*P-value (Conover)
Hippocampus	HFC	0.656 (0.615–0.691)	0.633 (0.608–0.663)	0.723 (0.687–0.774)	0.000006 (1, 3), (2, 3)
	GMV	0.319 (0.303–0.359)	0.292 (0.262–0.327)	0.241 (0.229–0.293)	0.00001 (1, 2, 3)
	WMV	0.239 (0.217–0.256)	0.222 (0.208–0.243)	0.205 (0.186–0.217)	0.001 (1, 3), (2, 3)
Insula	HFC	0.565 (0.538–0.586)	0.573 (0.547–0.587)	0.639 (0.608–0.657)	<0.000001 (1, 3), (2, 3)
	GMV	0.321 (0.291–0.353)	0.299 (0.285–0.319)	0.284 (0.255–0.295)	0.0003 (1, 3), (2, 3)
	WMV	0.236 (0.210–0.259)	0.224 (0.211–0.243)	0.210 (0.190–0.231)	0.011 (1, 3), (2, 3)
Precuneus	HFC	0.743 (0.694–0.805)	0.718 (0.655–0.758)	0.709 (0.659–0.796)	0.506
	GMV	0.282 (0.268–0.305)	0.273 (0.257–0.283)	0.260 (0.250–0.280)	0.031 (1, 3)
	WMV	0.177 (0.162–0.195)	0.169 (0.161–0.181)	0.153 (0.143–0.170)	0.002 (1, 3), (2, 3)
MTG	HFC	0.528 (0.492–0.545)	0.523 (0.493–0.540)	0.577 (0.534–0.585)	0.00004 (1, 3), (2, 3)
	GMV	0.300 (0.282–0.332)	0.277 (0.267–0.301)	0.268 (0.242–0.279)	0.0004 (1, 2, 3)
	WMV	0.202 (0.177–0.221)	0.187 (0.178–0.199)	0.170 (0.151–0.189)	0.001 (1, 3), (2, 3)

* *P-value by Kruskal–Wallis Test with Conover post-hoc test*.

*The specific regions-of-interest (ROI) areas were defined by the atlas-based areas of the hippocampus, insula, precuneus and middle temporal gyrus (MTG). Data are listed as median (25th percentile-75th percentile). Results of the post-hoc test in the parentheses are explained as the followings: significant difference among the three subject groups as (1, 2, 3), between CN and AD as (1, 3), between CN and MCI as (1, 2), and between MCI and AD (2, 3)*.

*CN, cognitively normal; MCI, mild cognitive impairment; AD, Alzheimer's disease; HFC, high-frequency conductivity; GMV, gray matter volume; WMV, white matter volume*.

#### Correlation With Age or MMSE Scores

Table 3 lists the results of the rank correlation analysis of the HFC, GMV, and WMV with age in each ROI and the results of the partial correlation analysis of those values with MMSE scores with adjustment for age. HFC values were positively correlated with age for insular, precuneus, and MTG ROIs. Both GMV and WMV were negatively correlated with age for all ROIs. HFC values were negatively correlated with MMSE scores for hippocampus, insular, and the MTG ROIs. Both GMV and WMV were positively correlated with MMSE scores for all ROIs.

**Table 3:** Results of correlation analyses of MRI measures with ages and Mini-Mental State Examination (MMSE) scores in the specific brain areas.

**ROI**	**Regressors**	**HFC**		**GMV**		**WMV**	
		**rho**	***p***	**rho**	***p***	**rho**	***p***
Hippocampus	Age	0.215	0.066	−0.270	0.020	−0.349	0.002
	*adjMMSE	−0.288	0.013	0.497	<0.0001	0.390	0.001
Insula	Age	0.397	0.001	−0.335	0.004	−0.319	0.006
	*adjMMSE	−0.408	0.0003	0.383	0.001	0.360	0.002
Precuneus	Age	0.316	0.006	0.349	0.002	−0.314	0.006
	*adjMMSE	0.098	0.412	0.317	0.006	0.383	0.001
MTG	Age	0.309	0.007	−0.367	0.001	−0.317	0.006
	*adjMMSE	−0.397	0.001	0.447	0.0001	0.414	0.0003

Data are listed as Spearman's coefficient (rho) by rank correlation coefficient with p-value, except ^*^adjMMSE which is the results of the partial correlation between MMSE scores and MRI measures with adjusting age.

*HFC, high-frequency conductivity; GMV, gray matter volume; WMV, white matter volume; ROI, regions-of-interest; MMSE, Mini-Mental State Examination; MTG, middle temporal gyrus*.

#### Receiver Operating Characteristic Curve Analysis

For the differentiation between the CN and AD groups, the largest AUC was the HFC value for the insular (*AUC* = 0.902, *p* < 0.0001) and followed by the GMV value for hippocampus (*AUC* = 0.880, *p* < 0.0001) and followed by the HFC value for hippocampus (*AUC* = 0.832, *p* < 0.0001) (Supplementary Table 6). The AUC values were reduced to differentiate the MCI group from the CN and AD groups for all three measurements. For the differentiation between the MCI and AD groups, the largest AUC was the HFC value for the insular (*AUC* = 0.889, *p* < 0.0001) and followed by the HFC value for hippocampus (*AUC* = 0.871, *p* < 0.0001) and followed by the HFC value for the middle temporal gyrus (*AUC* = 0.831, *p* < 0.0001) (Supplementary Table 6). Table 4 summarizes the results of the ROC curve analysis to evaluate adding the HFC to the GMV values for the group classifications. The addition of the HFC values slightly improved the classification between the subject groups. For the differentiation between the CN and AD groups, the largest AUC value was seen by adding the GMV of the hippocampus to the HFC of the insula (*AUC* = 0.928, *p* < 0.0001). For the differentiation between the MCI and AD groups, the largest AUC value was seen by adding the WMV of the hippocampus to the HFC of the insula (*AUC* = 0.894, *p* < 0.0001) or adding the three MRI measures together (*AUC* = 0.894, *p* < 0.0001).

**Table 4:** Results of receiver operating characteristic (ROC) curve analysis to evaluate the addition of high-frequency conductivity (HFC) index values to the GMV and WMV values for group classifications.

**MRI measures**	**CN vs. MCI**				**CN vs. AD**				**MCI vs. AD**			
	**SE**	**SP**	**AUC**	***p***	**SE**	**SP**	**AUC**	***p***	**SE**	**SP**	**AUC**	***p***
*Hippo GMV	55.56	83.33	0.701	0.007	79.17	82.61	0.880	<0.0001	56.52	85.19	0.721	0.003
*Hippo WMV	44.44	83.33	0.596	0.243	78.26	75.00	0.815	<0.0001	47.83	88.89	0.734	0.001
*Insular HFC	70.37	50.00	0.529	0.738	82.61	87.50	0.902	<0.0001	82.61	88.89	0.889	<0.0001
GMV+WMV	74.07	70.83	0.738	0.001	82.61	79.17	0.880	<0.0001	60.87	81.48	0.754	0.0003
GMV+ insular HFC	51.85	83.33	0.710	0.004	86.96	87.50	0.928	<0.0001	78.26	96.30	0.882	<0.0001
WMV+ insular HFC	44.44	83.33	0.590	0.271	82.61	95.83	0.911	<0.0001	82.61	92.59	0.894	<0.0001
GMV+WMV+ insular HFC	66.67	70.83	0.728	0.001	82.61	95.83	0.926	<0.0001	86.61	92.59	0.894	<0.0001

* *The receiver operating characteristic (ROC) analyses were performed using GMV at the hippocampus (Hippo), WMV at the hippocampus, and HFC at the insular. Added values of HFC index were evaluated combining between MRI measures*.

*HFC, high-frequency conductivity; GMV, gray matter volume; WMV, white matter volume; CN, cognitively normal; MCI, mild cognitive impairment; AD, Alzheimer's disease; SE, Sensitivity; SP, Specificity; AUC, Area under the ROC curve*.

## Discussion

### High-Frequency Conductivity Signals in AD

We obtained a good HFC map non-invasively and found that the HFC values were significantly increased with increasing disease severity (Table 2). Furthermore, the HFC value in the insula has a high AUC value to differentiate AD patients from the CN participants (*SE* = 82, *SP* = 97, *AUC* = 0.902, *p* < 0.0001), better than GMV in hippocampus (*SE* = 79, *SP* =83, *AUC* = 0.880, *p* < 0.0001) (Table 4, Supplementary Table 6). Finally, the results of the values added to the ROC curve analysis showed an improvement in the classification between the CN and AD groups using the GMV of the hippocampus and the HFC value of the insula (*SE* = 87, *SP* = 87, *AUC* = 0.928, *p* < 0.0001) (Table 4). The differentiation between MCI and AD was best using the HFC values of the insula (*SE* = 83, *SP* = 89, *AUC* = 0.889, *p* < 0.0001) (Supplementary Table 6). However, the differentiation between the CN and MCI groups was much less than between the CN and AD groups. Therefore, we need to further develop the HFC imaging sequence to use it for detecting MCI. Currently, we are developing a technique to map low-frequency conductivity in AD patients (23, 25). The diagnostic value of low-frequency conductivity is unknown.

The apparent electrical conductivity in the biological tissues is proportional to the summation of the multiplication of the ion concentrations and the electrical mobility of the charge carriers. From the HFC, which provides mixed information from the intracellular and extracellular compartments, decomposition techniques by separating the signals into the intracellular and extracellular compartments have the potential to recover the electrical properties in each separated compartment. In AD patients, both the concentration and diffusivity are increased by the demyelination and neurodegeneration of neurons replaced by CSF. Therefore, we expected increased conductivity in the AD patients compared to the CN participants. Furthermore, the previous studies showed increased metallic ions in AD patients caused by misfolding and aggregation of amyloid-beta proteins, which influenced homeostasis (5, 6) and imbalanced the Na+ and K+ ions (7, 8). These effects can be increased in HFC signals in AD patients.

### High-Frequency Conductivity Signals Correlated With MMSE Scores and Age

As we can see in Figure 3, HFC was strongly negatively correlated with the age adjusted MMSE scores, indicating that the HFC values increased with increasing disease severity. The neuronal loss in AD patients can cause an increase in ion concentrations and diffusivity in the regions, increasing the HFC. The previous studies suggested that Aβ could form aberrant ion channels in neuronal membranes (26) and increase ion concentrations (7). However, in this study, we did not verify the relationship between increasing HFC in AD patients and the accumulation of Aβ or tau proteins. Therefore, further studies are required to investigate the relationship. We found that the HFC values were significantly correlated with age and were increased with increasing age in some areas in the brain (Table 3). Since the GMV and WMV were decreased with increasing age, the neuronal loss with aging can cause an increase in HFC in the region.

## Conclusion

This study was the first-time application of the MREPT technique to diagnose AD patients by measuring HFC. We obtained a good HFC map non-invasively. We found that the HFC values were significantly increased in AD patients compared to CN people, revealing that neuronal degeneration in AD caused the increased HFC. The HFC values were increased with increasing age and increasing disease severity. The HFC values in the insula along with the GMV of the hippocampus can be used as an imaging biomarker to improve distinguishing AD from CN. These results might help in understanding AD pathophysiology, adding emphasis on the ion channel hypothesis. Further studies should be conducted to evaluate the direct relationship between neuronal loss and conductivity changes in the brain of patients with AD. In addition, this study was focused on the evaluation of HFC. However, it is also necessary to evaluate low-frequency conductivity in the brain of patients with AD.

## Data Availability Statement

The original contributions presented in the study are included in the article/Supplementary Material, further inquiries can be directed to the corresponding author/s.

## Ethics Statement

The studies involving human participants were reviewed and approved by Kyung Hee University Hospital at Gangdong Institutional Review Board. The patients/participants provided their written informed consent to participate in this study.

## Author Contributions

GHJ, OK, and ML contributed to conception and design of the study. SJ organized the database. ML performed the statistical analysis. SP wrote the first draft of the manuscript. HR, CWR, and ARC wrote sections of the manuscript. All authors contributed to manuscript revision, read, and approved the submitted version.

## Funding

The research was supported by the National Research Foundation of Korea (NRF) grants funded by the Ministry of Science and ICT (2020R1A2C1004749, GHJ; 2019R1A2C1004660, OK; 2020R1F1A1A01074353, ML), Republic of Korea.

## Conflict of Interest

The authors declare that the research was conducted in the absence of any commercial or financial relationships that could be construed as a potential conflict of interest.

## Publisher's Note

All claims expressed in this article are solely those of the authors and do not necessarily represent those of their affiliated organizations, or those of the publisher, the editors and the reviewers. Any product that may be evaluated in this article, or claim that may be made by its manufacturer, is not guaranteed or endorsed by the publisher.

## Abbreviations

3D T1W: three-dimensional (3D) T1-weighted (T1W)
AD: Alzheimer's disease
CDR: clinical dementia rating
CN: cognitively normal
GMV: gray matter volume
HFC: high frequency conductivity
MCI: mild cognitive impairment
MMSE: mini-mental state examination
MREPT: MRI-based electrical property tomography
ROI: region-of-interest
WMV: white matter volume.
