# Supplementary material for: Application of High-Frequency Conductivity Map Using MRI to Evaluate It in the Brain of Alzheimer's Disease Patients
Source: Front Neurol. 2022 May 16;13:872878. doi: 10.3389/fneur.2022.872878 (PMC9150564; doi:10.3389/fneur.2022.872878)
Supplement: Supplementary file 1 [file Table_1.docx]

**Application of High-Frequency Conductivity Map Using MRI to Evaluate It in the Brain of Alzheimer’s Disease Patients**

**Supplementary Materials and Methods**

**Reconstruction of the HFC map in the brain**

The electrical conductivity of biological tissue as a function of frequency is complicated by the anisotropic nature of the tissue, the non-homogeneous nature of the extracellular and intracellular compartments, and randomly distributed cell sizes. At the Larmor frequency of 128 MHz at 3T, HFC provides combined information on the concentration and mobility of ions in the extracellular and intracellular compartments. The relationship between the B1 field denoted as $\mathbf{B}_{1}$ and the electrical properties are expressed as:

$\nabla^{2}\mathbf{B}_{1}=i\omega\mu_{0} \tau_{H}\mathbf{B}_{\mathbf{1}} -\frac{\nabla\tau_{H}}{\tau_{H}}\times\left( \nabla\times\mathbf{B}_{1} \right)$ [1]

where ω is the angular frequency, $\mu_{0}=4\pi\times{10}^{-7}N/A^{2}$is the magnetic permeability of the free space, and $\tau_{H}=\sigma_{H}+i\omega\epsilon_{H}$ at high-frequency conductivity $\sigma_{H}$ and permittivity $\epsilon_{H}$ (14)(10). The transverse field of $\mathbf{B}_{1}$ can be decomposed into the positively rotating field $B_{1}^{+}=\frac{1}{2}\left( B_{x}+iB_{y} \right)$ and the negatively rotating field $B_{1}^{-}=\frac{1}{2}\left( B_{x}-iB_{y} \right).$ With the conventional MRI scanner having a single transmit channel, the magnetic field $B_{1}^{+}$ component is available. We denoted $\phi^{+}$ and $\phi^{-}$ as the phase terms of $B_{1}^{+}$and $B_{1}^{-}$, respectively. By assuming $\sigma_{H}\gg\omega\epsilon_{H}$, a phase-based MREPT formula was derived as:

$\left( \nabla\phi^{\mathrm{tr}}\cdot\nabla\left( \frac{1}{\sigma_{H}} \right) \right)+\frac{\nabla^{2}\phi^{\mathrm{tr}}}{\sigma_{H}}-2\omega\mu_{0}=0$ [2]

where $\phi^{\mathrm{tr}}=\phi^{+}+\phi^{-}$. To stabilize the formula [2], the MREPT formula based on a convection reaction equation can be derived by adding the regularization coefficient *c* (23)(16):

$-c\text{∇}^{\text{2}}\left( \frac{1}{\sigma_{H}} \right)+\left( \text{∇}\phi^{\mathrm{tr}}\cdot\nabla\left( \frac{1}{\sigma_{H}} \right) \right)+\frac{\text{∇}^{\text{2}}\phi^{\mathrm{tr}}}{\sigma_{H}}=2\omega\mu_{0}$ [3]

MREPT depends upon the relatively weak phase signal by a secondary RF magnetic field from the induced electrical current by the time-varying RF field. Due to the weak phase signal and noise artifacts, a multi-echo spin-echo MR pulse sequence is advantageous to reduce the noise artifacts using the weight for *k*th echo:

$$\phi^{\mathrm{tr}}=\sum_{k=1}^{N_{E}} w_{k}\phi^{k}, w_{k}=\frac{\left| \rho_{k} \right|^{2}}{\sum_{j=1}^{N_{E}} \left| \rho_{j} \right|^{2}}$$

where $\phi^{k}$ and $\rho_{k}$ are the phase signal and complex MR signal, respectively, for *k*th echo. To solve the convection reaction partial differential equation in [3], we used the 2-dimensional finite-difference method. For each image matrix, the equation [3] is written as:

$\left[ \begin{matrix} \vdots\\ -c\left( \frac{\partial^{2}}{\partial x^{2}}+\frac{\partial^{2}}{\partial y^{2}} \right)+\frac{\partial\phi^{\mathrm{tr}}}{\partial x}\frac{\partial}{\partial x}+\frac{\partial\phi^{\mathrm{tr}}}{\partial y}\frac{\partial}{\partial y}+\frac{\partial^{2}\phi^{\mathrm{tr}}}{\partial x^{2}}+\frac{\partial^{2}\phi^{\mathrm{tr}}}{\partial y^{2}} \\ \vdots\end{matrix} \right]\left[ \begin{matrix} \vdots\\ \frac{1}{\sigma_{H}} \\ \vdots\end{matrix} \right]=\left[ \begin{matrix} \vdots\\ 2\omega\mu_{0} \\ \vdots\end{matrix} \right]$ [4]

The finite-difference formulations at each grid point $\left( x_{i},y_{j} \right), i=1,\cdots,n_{x}, j=1,\cdots,n_{y}$ are as follows:

$\frac{\partial u_{i,j}}{\partial x}=\frac{u_{i,j+1}-u_{i,j-1}}{h}$, $\frac{\partial u_{i,j}}{\partial y}=\frac{u_{i+1,j}-u_{i-1,j}}{h}$ and $\frac{\partial^{2}u_{i,j}}{\partial x^{2}}+\frac{\partial^{2}u_{i,j}}{\partial y^{2}}=\frac{u_{i+1,j}+u_{i-1,j}+u_{i,j+1}+u_{i,j-1}-4u_{i,j}}{h^{2}}$

where $u_{i,j}$ is the value $\frac{1}{\sigma_{H}}$ at $\left( x_{i},y_{j} \right)$ and h is the voxel size in the image slice. The finite-difference method for solving the equation [4] is to find the solutions of a linear matrix system $\mathbf{Au}= \mathbf{b}$, where $\boldsymbol{A\in}\mathbb{(R}^{n_{x}\times n_{y}}\boldsymbol{,}\mathbb{R}^{n_{x}\times n_{y}}$**)**, $\mathbf{u}\boldsymbol{\in}\mathbb{R}^{n_{x}\times n_{y}}\boldsymbol{,}$and $\mathbf{b}\boldsymbol{\in}\mathbb{R}^{n_{x}\times n_{y}}$ with the appropriate processing of the Dirichlet boundary conditions. We modified the linear equations and solved the equations at the interior nodes to estimate the conductivity values at the interior nodes belonging to the brain region. We selected the boundary nodes of the brain mask and assigned a reference conductivity value to the boundary nodes. At the non-boundary nodes, we set the elements of $\mathbf{u}$ to 0 and replaced $\mathbf{b}$ with $\mathbf{b- Au}$. The solutions at the interior nodes can be obtained by solving the equation:

$\mathbf{A(}interior nodes, interior nodes\mathbf{)u(}interior nodes\mathbf{)}= \mathbf{b(}interior nodes\mathbf{)}$ [5]

The matrix equation of [5] can be solved using the symmetricity and sparsity properties of $\mathbf{A}$**.**

**Supplementary Result Tables**

**Supplementary Table S1. Areas of significant differences of HFC among the three participant groups using the voxel-based full factorial one-way analysis of covariance test**

| Group analysis | Cluster size | Cluster location | BA | Talairach coordinates | Z score | ROI |  |
| --- | --- | --- | --- | --- | --- | --- | --- |
| CN<AD |  | | | | | | |
|  | 302880 | Lt Superior Temporal Gyrus WM |  | -55.56, -49.27, 20.74 | 6.787 |  |  |
|  |  | Rt Frontal Sub-Gyral Gyrus WM |  | 40.43, 6.65, 22.26 | 6.537 |  |  |
|  |  | Rt Frontal Precentral Gyrus WM |  | 37.54, 1.69, 29.85 | 6.470 |  |  |
| MCI<AD |  | | | | | | |

|  | 283428 | Lt Temporal Supramarginal Gyrus WM |  | -55.58, -48, 22.21 | 6.846 |  |
| --- | --- | --- | --- | --- | --- | --- |
|  |  | Rt Frontal Sub-Gyral WM |  | 40.43, 6.65, 22.26 | 6.576 |  |
|  |  | Rt Sub-lobar Insula GM | 13 | 42.03, -6.9, 2.08 | 6.576 | ROI |

We listed the significant locations with false discovery rate (FDR) P = 0.05 with at least 100 contiguous cluster voxels. Age was used as covariate.

CN, cognitively normal; MCI, mild cognitive impairment; AD, Alzheimer’s disease; Rt, right; Lt, left; BA, Brodmann area; GM, Gray Matter; WM, white matter; HFC, high frequency conductivity; ROI, region-of-interest; MTG, Middle Temporal Gyrus

**Supplementary Table S2. Areas of significant differences of GMV among the three participant groups using the voxel-based full factorial one-way analysis of covariance test**

| Group analysis | Cluster size | | | | Cluster location | BA | Talairach coordinates | Z score | ROI |  |
| --- | --- | --- | --- | --- | --- | --- | --- | --- | --- | --- |
| CN>MCI | | | |  | | | | | | |
|  | 116953 | | | | Lt Frontal Subcallosal Gyrus | 34 | -11.87, 2.91, -14.57 | 5.447 |  |  |
|  |  | | | | Rt Superior Frontal Gyrus | 10 | 21.27, 56.29, 15.37 | 5.140 |  |  |
|  |  | | | | Lt Inferior Temporal Gyrus | 20 | -46.41, -8.04, -31.05 | 5.098 |  |  |
|  | 752 | | | | Rt Occipital Cuneus |  | 11.02, -75.06, 17.62 | 3.617 |  |  |
|  |  | | | | Rt Limbic Posterior Cingulate | 30 | 8.33, -51.16, 18.49 | 2.659 |  |  |
|  | 2480 | | | | Rt Middle Temporal Gyrus | 39 | 51.24, -64.61, 24.7 | 3.543 | ROI |  |
|  |  | | | | Rt Superior Temporal Gyrus | 39 | 50.09, -53.25, 9.54 | 3.502 |  |  |
|  | 217 | | | | Lt Superior Parietal Lobule | 7 | -36.61, -71.22, 52.31 | 3.456 |  |  |
|  | 319 | | | | Lt Posterior Cerebellar Tonsil |  | -32.57, -49.52, -40.15 | 3.246 |  |  |
|  | 139 | | | | Rt Limbic Posterior Cingulate | 30 | 30.59, -70.05, 8.97 | 3.242 |  |  |
|  | 177 | | | | Lt Temporal Fusiform Gyrus | 37 | -48.13, -43.4, -15.51 | 3.113 |  |  |
|  | 352 | | | | Lt Occipital Middle Temporal Gyrus | 19 | -56.93, -63.27, 14.89 | 3.081 |  |  |
|  |  | | | | Lt Middle Temporal Gyrus | 39 | -48.56, -75.36, 8.48 | 2.335 | ROI |  |
|  | 157 | | | | Rt Superior Frontal Gyrus | 8 | 8.28, 44.44, 50.52 | 3.064 |  |  |
|  | 354 | | | | Rt Inferior Parietal Lobule | 40 | 49.85, -39.58, 32.55 | 3.062 |  |  |
|  | 630 | | | | Lt Occipital Cuneus |  | -12.55, -77.47, 14.29 | 3.051 |  |  |
|  |  | | | | Lt Middle Occipital Gyrus | 19 | -33.39, -78.89, 15.16 | 3.017 |  |  |
|  | 102 | | | | Lt Parietal Postcentral Gyrus | 7 | -9.02, -54.77, 70.55 | 3.024 |  |  |
|  | 725 | | | | Rt Parietal Precuneus | 7 | 7.87, -54.55, 53.29 | 2.925 | ROI |  |
|  |  | | | | Rt Superior Parietal Lobule | 7 | 7.79, -63.33, 56.51 | 2.617 |  |  |
|  | 128 | | | | Rt Superior Parietal Lobule | 7 | -21.47, -72.22, 61.93 | 2.894 |  |  |
|  | 223 | | | | Rt Middle Frontal Gyrus | 46 | 46.05, 28.56, 23.98 | 2.808 |  |  |
|  | 145 | | | | Rt Frontal Precentral Gyrus | 4 | 56.99, -8.35, 26.07 | 2.545 |  |  |
| CN>AD | |  | | | | | | | | |
|  | 395925 | | | | Lt Frontal Subcallosal Gyrus | 34 | -21.64, 3.97, -10.58 | 7.624 |  |  |
|  |  | | | | Rt Sub-lobar Insula | 13 | 36.36, 1.45, 13.14 | 7.355 | ROI |  |
|  |  | | | | Rt Temporal Sub-Gyral | 21 | 39.35, -12.11, -7.01 | 7.346 |  |  |
|  | 122 | | | | Lt Superior Frontal Gyrus | 6 | -8.82, 0.12, 71.7 | 2.318 |  |  |
| MCI>AD | | |  | | | | | | |  |
|  | 214136 | | | | Rt Sub-lobar Insula | 13 | 37.75, 1.44, 13.16 | 6.259 | ROI | |
|  |  | | | | Rt Middle Temporal Gyrus | 21 | 57.23, -29.89, 1.06 | 5.922 | ROI | |
|  |  | | | | Rt Superior Temporal Gyrus | 41 | 48.82, -27.7, 7.88 | 5.408 |  | |
|  | 2976 | | | | Lt Posterior Inferior Semi-Lunar Lobule |  | -42.31, -66.1, -43.24 | 3.233 |  | |
|  |  | | | | Lt Posterior Pyramis |  | -34.12, -72.65, -34.26 | 2.489 |  | |
|  | 177 | | | | Lt Posterior Cerebellar Tonsil |  | -13.08, -34.11, -39.71 | 3.135 |  | |
|  | 290 | | | | Lt Parietal Postcentral Gyrus | 7 | -24.21, -52.77, 65.08 | 2.960 |  | |
|  | 131 | | | | Rt Occipital Lingual Gyrus | 17 | 15.54, -98.5, -16.95 | 2.383 |  | |

We listed the significant locations with false discovery rate (FDR) P = 0.05 with at least 100 contiguous cluster voxels. Age and total intracranial volume (TIV) were used as covariates.

CN, cognitively normal; MCI, mild cognitive impairment; AD, Alzheimer’s disease; Rt, right; Lt, left; BA, Brodmann area; GM, Gray Matter; WM, White Matter; CSF, Cerebrospinal Fluid; GMV, gray matter volume; ROI, region-of-interest; MTG, Middle Temporal Gyrus; AC, anterior cingulate

**Supplementary Table S3. Areas of significant differences of WMV among the three participant groups using the voxel-based full factorial one-way analysis of covariance test**

| Group analysis | Cluster size | | | Cluster location | BA | Talairach coordinates | Z score | ROI |  |
| --- | --- | --- | --- | --- | --- | --- | --- | --- | --- |
| CN>AD | |  | | | | | | | |
|  | 370566 | | | Rt Temporal Caudate tail GM |  | 36.5, -27.73, -5.84 | 65535 |  |  |
|  |  | | | Rt Sub-lobar Extra-Nuclear |  | 19.52, -2.57, 25.98 | 65535 |  |  |
|  |  | | | Lt Sub-lobar Lateral Ventricle CSF |  | -35.74, -34.6, -5.01 | 65535 |  |  |
| MCI>AD | | |  | | | | | | |

|  | 318004 | Rt Sub-lobar Extra-Nuclear |  | 19.52, -2.57, 25.98 | 65535 |  |
| --- | --- | --- | --- | --- | --- | --- |
|  |  | Rt Temporal Sub-Gyral |  | 37.88, -30.53, -6.08 | 65535 |  |
|  |  | Rt Sub-lobar Insula |  | 32.07, -6.3, 20.44 | 65535 | ROI |

We listed the significant locations with false discovery rate (FDR) P = 0.05 with at least 100 contiguous cluster voxels. Age and total intracranial volume (TIV) were used as covariates.

CN, cognitively normal; MCI, mild cognitive impairment; AD, Alzheimer’s disease; Rt, right; Lt, left; BA, Brodmann area; WM, White Matter; WMV, white matter volume; ROI, region-of-interest; CSF, Cerebrospinal Fluid

**Supplementary Table S4. Areas of significant associations of between MRI measures and ages for the three participant groups using the voxel-based multiple regression test.**

| Group analysis | Cluster size | Cluster location | BA | Talairach coordinates | Z score | ROI |
| --- | --- | --- | --- | --- | --- | --- |
| HFC (+)age | | | | | | |
|  | 3304 | Lt Limbic Cingulate Gyrus WM |  | -12.52, -23.33, 27.98 | 4.928 |  |
|  |  | Lt Frontal Sub-Gyral WM |  | -19.17, 21.33, 18.59 | 4.625 |  |
|  |  | Limbic Cingulate Gyrus WM |  | -11.31, -32.9, 39.26 | 3.906 |  |
|  | 364 | Lt Parietal Precuneus WM |  | -11.31, -52.07, 33.39 | 4.448 |  |
|  | 312 | Rt Inferior Frontal Gyrus |  | 49.03, 16.82, 4.45 | 3.975 |  |
|  | 163 | Lt Occipital Cuneus |  | -8.62, -74.71, 33.99 | 3.677 |  |
| GMV (-)age | | | | | | |
|  | 9467 | Rt Posterior Cerebellar Tonsil |  | 9.14, -38.29, -41.09 | 4.349 |  |
|  |  | Rt Posterior Cerebellar Tonsil |  | 45.08, -49.18, -32.05 | 4.290 |  |
|  | 1623 | Lt Frontal Precentral Gyrus | 4 | -29.44, -27.03, 47.16 | 4.340 |  |
|  | 3752 | Lt Limbic Parahippocampal Gyrus | 19 | -20.57, -54.65, -2.6 | 4.179 |  |
|  |  | Rt Limbic Parahippocampal Gyrus | 19 | 19.7, -56.11, -3.41 | 3.884 |  |
|  |  | Rt Occipital Lingual Gyrus | 18 | 8.54, -70.16, -3.58 | 3.503 |  |
|  | 1752 | Lt Parietal Precuneus | 7 | -18.46, -63.68, 46.58 | 3.926 | ROI |
|  |  | Lt Middle Occipital Gyrus | 18 | -26.36, -79.67, 8.44 | 3.759 |  |
|  | 290 | Lt Medial Frontal Gyrus | 10 | -14.78, 51.28, 10.24 | 3.865 |  |
|  | 1881 | Lt Sub-lobar Insula | 13 | -40.24, -24.75, 24.22 | 3.760 | ROI |
|  |  | Lt Transverse Temporal Gyrus | 42 | -58.07, -14.83, 10 | 3.017 |  |
|  | 225 | Lt Occipital Lingual Gyrus | 18 | -13.71, -84.02, -5.27 | 3.635 |  |
|  | 117 | Lt Limbic Cingulate Gyrus | 32 | -12.32, 29.74, 28.51 | 3.554 |  |
|  | 160 | Lt Middle Temporal Gyrus | 21 | -50.84, -18.24, -13.17 | 3.470 | ROI |
|  | 564 | Lt Frontal Paracentral Lobule | 6 | -7.24, -24.48, 49.13 | 3.470 |  |
|  | 966 | Lt Parietal Precuneus | 7 | -1.7, -47.87, 42.95 | 3.453 | ROI |
|  |  | Lt Limbic Cingulate Gyrus | 31 | -11.29, -41.45, 35.29 | 3.290 |  |
|  | 263 | Lt Superior Frontal Gyrus | 9 | -16.49, 40.68, 32.18 | 3.433 |  |
|  |  | Lt Medial Frontal Gyrus | 8 | -13.89, 28.44, 41.87 | 3.283 |  |
|  | 107 | Lt Sub-lobar Thalamus |  | -2.62, -8.79, 18.26 | 3.396 |  |
|  | 137 | Lt Middle Temporal Gyrus | 21 | -39.51, -1.49, -26.26 | 3.371 | ROI |
|  | 340 | Rt Sub-lobar Insula | 13 | 36.25, -22.7, 14.9 | 3.346 | ROI |
|  | 105 | Rt Middle Frontal Gyrus | 6 | 24.8, -8.75, 45.76 | 3.323 |  |
|  | 282 | Lt Posterior Cerebellar Tonsil |  | -36.78, -56.74, -38.21 | 3.194 |  |
|  | 137 | Rt Frontal Subcallosal Gyrus | 25 | 10.38, 23.63, -10.88 | 3.124 |  |
|  | 122 | Lt Superior Temporal Gyrus | 22 | -52.38, -2.9, 3.12 | 3.083 |  |

Results are shown the positive (+) or negative (-) association with age. There is no significant association between WMV and Age. HFC, GMV and WMV are listed the significant locations with false discovery rate (FDR) P = 0.05 with at least 100 contiguous cluster voxels.

Rt, right; Lt, left; BA, Brodmann area; GM, Gray Matter; WM, White Matter; HFC, high-frequency conductivity; GMV, gray matter volume (GMV); WMV, white matter volume; ROI, region-of-interest; MTG, Middle Temporal Gyrus

**Supplementary Table S5.** **Areas of significant associations of between MRI measures and MMSE scores for the three participant groups using the voxel-based multiple regression test**

| Group analysis | Cluster size | | Cluster location | BA | Talairach coordinates | Z score | ROI |  |  |
| --- | --- | --- | --- | --- | --- | --- | --- | --- | --- |
| HFC (-)MMSE | | | | | | | | |  |
|  | | 135655 | Rt Frontal Precentral Gyrus GM | 6 | 36.01, -3.41, 38.8 | 4.755 |  |  |  |
|  |  |  | Rt Postcentral Gyrus | 3 | 37.34, -21.71, 38.44 | 4.500 |  |  |  |
|  |  |  | Rt Inferior Frontal Gyrus | * | 44.46, 0.13, 31.17 | 4.418 |  |  |  |
|  |  | 195 | Rt Occipital Lingual Gyrus | * | 23.73, -89.51, -3.35 | 2.895 |  |  |  |
|  |  | 225 | Rt Parietal Sub-Gyral | * | 24.64, -35.53, 50.42 | 2.819 |  |  |  |
| GMV (+)MMSE | | | | | | | | | |
|  | 169939 | | Rt Limbic Parahippocampal Gyrus | 27 | 19.8, -29.3, -3.57 | 4.875 |  |  |  |
|  |  | | Rt Middle Temporal Gyrus | 22 | 54.4, -35.71, 3.16 | 4.397 | ROI |  |  |
|  |  | | Rt Lateral Globus Pallidus |  | 25.45, -12.17, -5.9 | 4.318 |  |  |  |
|  | 1998 | | Rt Frontal Precentral Gyrus | 9 | 40.24, 4.79, 36.49 | 3.805 |  |  |  |
|  |  | | Rt Middle Frontal Gyrus | 6 | 35.88, 15.69, 55.02 | 2.900 |  |  |  |
|  | 356 | | Lt Middle Frontal Gyrus | 6 | -52.85, 11.48, 43.66 | 3.107 |  |  |  |
|  | 321 | | Lt Parietal Postcentral Gyrus | 7 | -27.02, -53.02, 67.71 | 3.067 |  |  |  |
|  | 201 | | Lt Posterior Cerebellar Tonsil |  | -13.08, -34.11, -39.71 | 2.706 |  |  |  |
|  | 228 | | Rt Parietal Postcentral Gyrus | 5 | 25.77, -43.38, 68.17 | 2.686 |  |  |  |
|  | 237 | | Rt Superior Parietal Lobule | 7 | 20.23, -65.19, 60.6 | 2.593 |  |  |  |
|  | 126 | | Lt Middle Frontal Gyrus | 46 | -45.29, 44.84, 5.06 | 2.449 |  |  |  |
|  | 103 | | Rt Frontal Sub-Gyral | 6 | 26.11, -3.82, 53 | 2.448 |  |  |  |
|  | 131 | | Lt Superior Frontal Gyrus | 6 | -18.32, 16.85, 59.61 | 2.422 |  |  |  |
| WMV (+)MMSE | | | | | | | | | |
|  | 115280 | | Lt Frontal Sub-Gyral |  | -24.75, 20.67, 20.68 | 4.417 |  |  |  |
|  |  | | Rt Temporal sub-Gyral |  | 42.12, -28.63, -11.24 | 4.234 |  |  |  |
|  |  | | Rt Middle Temporal Gyrus |  | 54.53, -37.47, -7.81 | 4.096 | ROI |  |  |
|  | 681 | | Lt Occipital Cuneus |  | -11.31, -90.97, 22.49 | 3.221 |  |  |  |
|  | 146 | | Rt Medial Frontal Gyrus |  | 17.31, 48.22, -3.02 | 2.499 |  |  |  |
|  | 186 | | Rt Posterior Cerebellar Tonsil |  | 29.95, -42.59, -41.14 | 2.489 |  |  |  |
|  |  | |  |  |  |  |  |  |  |

Results are shown the positive (+) or negative (-) association with MMSE scores. HFC, GMV, and WMV are listed the significant locations with the false discovery rate (FDR) P = 0.05 with at least 100 contiguous cluster voxels.

Rt, right; Lt, left; BA, Brodmann area; GM, Gray Matter; HFC, high-frequency conductivity; GMV, gray matter volume (GMV); WMV, white matter volume; MMSE, Mini-Mental State Examination; ROI, region-of-interest; MTG, Middle Temporal Gyrus

**Supplementary Table S6. Results of a receiver operating characteristic (ROC) curve analysis of MRI measures obtained in the specific brain areas.**

| ROI | | CN vs MCI | | | | CN vs AD | | | | MCI vs AD | | | |
| --- | --- | --- | --- | --- | --- | --- | --- | --- | --- | --- | --- | --- | --- |
|  |  | SE | SP | AUC | p | SE | SP | AUC | p | SE | SP | AUC | p |
| Hippocampus | HFC | 74.07 | 50.00 | 0.585 | 0.305 | 73.91 | 83.33 | 0.832 | <0.0001 | 73.91 | 92.59 | 0.871 | <0.0001 |
|  | GMV | 55.56 | 83.33 | 0.701 | 0.007 | 79.17 | 82.61 | 0.880 | <0.0001 | 56.52 | 85.19 | 0.721 | 0.003 |
|  | WMV | 44.44 | 83.33 | 0.596 | 0.243 | 78.26 | 75.00 | 0.815 | <0.0001 | 47.83 | 88.89 | 0.734 | 0.001 |
| Insular | HFC | 70.37 | 50.00 | 0.529 | 0.738 | 82.61 | 87.50 | 0.902 | <0.0001 | 82.61 | 88.89 | 0.889 | <0.0001 |
|  | GMV | 88.89 | 50.00 | 0.647 | 0.074 | 82.61 | 70.83 | 0.810 | <0.0001 | 73.91 | 62.96 | 0.739 | 0.001 |
|  | WMV | 77.78 | 45.83 | 0.554 | 0.525 | 82.61 | 58.33 | 0.732 | 0.002 | 47.83 | 92.59 | 0.700 | 0.008 |
| Precuneus | HFC | 66.67 | 58.33 | 0.594 | 0.248 | 65.22 | 58.33 | 0.567 | 0.444 | 34.78 | 81.48 | 0.522 | 0.797 |
|  | GMV | 66.67 | 62.50 | 0.634 | 0.093 | 60.87 | 75.00 | 0.721 | 0.003 | 69.57 | 51.85 | 0.588 | 0.288 |
|  | WMV | 62.96 | 66.67 | 0.573 | 0.388 | 65.22 | 83.33 | 0.766 | 0.0002 | 60.87 | 92.59 | 0.736 | 0.002 |
| MTG | HFC | 62.96 | 54.17 | 0.503 | 0.971 | 65.22 | 91.67 | 0.824 | <0.0001 | 65.22 | 88.89 | 0.831 | <0.0001 |
|  | GMV | 51.85 | 83.33 | 0.670 | 0.031 | 82.61 | 79.17 | 0.813 | <0.0001 | 43.48 | 88.89 | 0.694 | 0.011 |
|  | WMV | 77.78 | 54.17 | 0.582 | 0.336 | 60.87 | 83.33 | 0.790 | <0.0001 | 52.17 | 96.30 | 0.752 | 0.001 |

The specific ROI areas were defined by the atlas-based areas of the hippocampus,insular, precuneus and middle temporal gyrus (MTG).

CN, cognitively normal; MCI, mild cognitive impairment; AD, Alzheimer’s disease; SE, Sensitivity; SP, Specificity; AUC, Area under the ROC curve; HFC, high-frequency conductivity; GMV, gray matter volume; WMV, white matter volume; ROI, region-of-interest
